# Mobilisation and dysfunction of haematopoietic stem/progenitor cells after *Listonella anguillarum* infection in ayu, *Plecoglossus altivelis*

**DOI:** 10.1038/srep28082

**Published:** 2016-06-16

**Authors:** Xin-Jiang Lu, Qiang Chen, Ye-Jing Rong, Jiong Chen

**Affiliations:** 1 Laboratory of Biochemistry and Molecular Biology, School of Marine Sciences, Ningbo University, Ningbo 315211, China; 2 Collaborative Innovation Center for Zhejiang Marine High-efficiency and Healthy Aquaculture, Ningbo University, Ningbo 315211, China

## Abstract

Haematopoietic stem/progenitor cells (HSPCs) can mobilise into blood and produce immune cell lineages following stress. However, the homeostasis and function of HSPCs after infection in teleosts are less well known. Here, we report that *Listonella anguillarum* infection enhances HSPC mobilisation and reduces their differentiation into myeloid cells in ayu (*Plecoglossus altivelis*), an aquacultured teleost in East Asia. We established a colony-forming unit culture (CFU-C) assay to measure HSPCs using conditioned medium from peripheral blood mononuclear cells stimulated with phytohaemagglutinin. The number of CFU-Cs decreased in the head kidney and increased in the blood and spleen of ayu infected with *L. anguillarum*. HSPC mobilisation after *L. anguillarum* infection was mediated by norepinephrine. Furthermore, HSPCs from ayu treated with *L. anguillarum* lipopolysaccharide (LPS) showed defective myeloid differentiation and could no longer rescue *L. anguillarum-*infected ayu. HSPC expansion was suppressed after *L. anguillarum* infection or its LPS treatment *in vitro*. These results reveal a link between HSPC regulation and pathogen infection in teleosts.

Haematopoietic stem cells (HSCs) give rise to all cells of lymphoid and myeloerythroid lineages^1^. In mammals, haematopoiesis begins in the yolk sac of the embryo and then shifts to the placenta, foetal liver, and adult bone marrow. Haematopoietic stem/progenitor cells (HSPCs) in the bone marrow remain in the dormant stage^2^. Stress conditions due to bleeding, infection, or DNA damage can impact HSPC mobilisation into the blood^3^. HSPCs have a strong self-renewal capability to produce progeny with the same developmental potential^4^. Injection of a single HSC from the bone marrow into a lethally irradiated animal can rescue the entire immune system^5^. Although several studies have reported the mechanisms underlying HSPC homeostasis, self-renewal, and differentiation in mammals, the process and mechanism of HSPC mobilisation and function in teleosts are less well known.

Systemically infected host organisms utilise HSPCs to generate an immune response and maintain homeostatic conditions in the blood^6^. HSPC responses against infection are pathogen-specific. In response to a systemic infection, such as with *Escherichia coli*, HSPCs expand, mobilise, and differentiate^7^. However, some pathogens, such as *Ehrlichia chaffeensis* or *Anaplasma phagocytophilum*, reduce HSPC numbers and the cellularity of bone marrow^8^,^9^. Several pathways have been found to mediate the effect of infection on HSPCs. TNFα is a major pro-inflammatory cytokine first identified as a serum-derived factor upon infection. TNFα has been classically understood to inhibit growth of HSPCs^10^. In addition to the indirect effect of pathogens on HSPCs via TNFα, HSPCs may directly sense pathogens. Human HSPCs express several Toll-like receptors^11^, which are the most common receptors for lipopolysaccharide (LPS), a strong inflammatory agent from the bacterial wall. Furthermore, the stimulatory cytokine granulocyte colony-stimulating factor (G-CSF) is elevated in response to *E. coli* infection^12^. Because G-CSF is widely used as an HSPC-mobilising agent^13^, it may mediate infection-induced HSPC mobilisation.

Haematopoiesis in teleosts is similar to that in mammals, as demonstrated by several investigations in zebrafish (*Danio rerio*)^14^, goldfish (*Carassius auratus* L.)^15^,^16^ and carp (*Cyprinus carpio*)^17^. The zebrafish has recently emerged as a powerful genetic model of haematopoiesis^18^,^19^. However, owing to their small body size, *in vivo* functional analysis of haematopoietic cells in zebrafish is difficult. Teleosts possess both adaptive and innate immune systems, which are similar to, and in some cases different from, mammalian immune system^20^. Some pattern-recognizing receptors do not exist in teleosts, such as CD14, a receptor important for LPS binding^21^. To understand the diversity of immune mechanisms in vertebrates, it is important to consider the benefits of the immune system for infection control in teleosts and also helpful to understand the system’s evolution in vertebrates^22^. Hence, more investigation is needed to elucidate HSPC homeostasis in teleosts after infection with its pathogens.

The ayu, *Plecoglossus altivelis,* is an economically important fish in East Asian countries. Bacterial diseases caused by *Listonella anguillarum* have been a major cause of losses in the ayu culture industry^23^. Considering the important role of HSPCs in the immune response, we investigated HSPC homeostasis and function after *L. anguillarum* infection. First, we established a method to assay the colony-forming units of HSPCs in ayu tissues and blood. Then, the number of HSPCs in the tissues and blood after *L. anguillarum* infection was determined. In addition, the differentiation capability of HSPCs from *L. anguillarum* LPS*-*infected ayu was analysed.

## Results

### Separation and characterisation of HSPCs in the ayu

**Figure 1:**
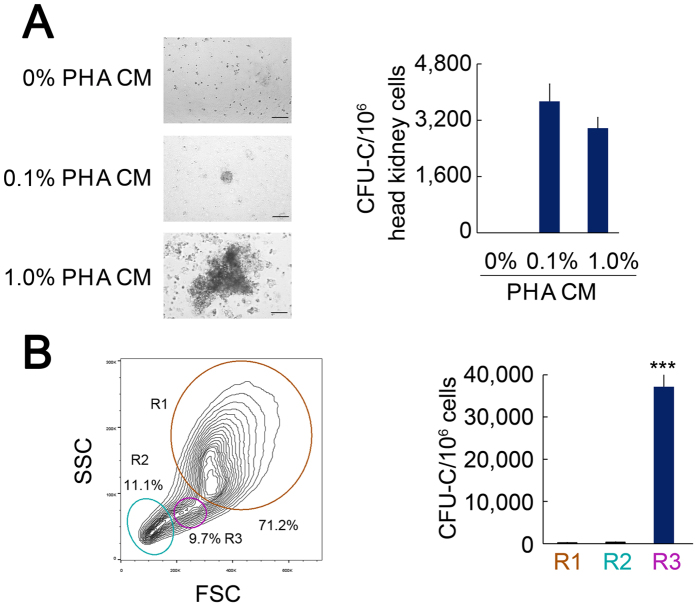
Separation and identification of head kidney-derived HSPCs. (**A**) PHA CM stimulates colony formation in methylcellulose cultures. (**B**) Separation of major cell lineages by light-scatter characteristics. FSC is directly proportional to cell size, and SSC is indicative of cellular granularity. FSC^hi^SSC^int^ subsets are composed of HSPC-enriched cells (R3). The data are expressed as the means ± SEM; ***p < 0.001; n = 5.

We employed conditioned medium from peripheral blood mononuclear cells (PBMC) stimulated with phytohaemagglutinin (PHA CM) to detect HSPCs in the head kidney of the ayu. Treatment with 0.1% and 1% PHA CM produced the colony-forming unit-culture (CFU-C) (Fig. 1A). A total of 3717 CFU-Cs induced by 0.1% PHA CM per 10^6^ head kidney mononuclear cells (MNCs) were produced. We used flow cytometry to detect HSPCs in the head kidney. Head kidney MNCs were analysed by flow cytometry using light-scatter characteristics as previously described^14^. Forward scatter (FSC) is directly proportional to cell size, and side scatter (SSC) is proportional to cellular granularity. Using combined scatter profiles, we separated the major cell lineages from the head kidney MNCs. On the basis of previous flow cytometry-based separation of major blood cell lineages from the zebrafish kidney^14^, the separations in this study were as follows: myelomonocytic cells, mainly including neutrophils, monocytes, and macrophages, were in an FSC^hi^SSC^hi^ population (R1); lymphoid cells were enriched in an FSC^lo^SSC^lo^ subset (R2); and HSPC-enriched cells were in an FSC^hi^SSC^int^ subset (R3, Fig. 1B). The numbers of CFU-Cs in gates R1, R2, and R3 were 234, 378, and 37,038, respectively (Fig. 1B), suggesting that R3 is an HSPC-enriched cell subset.

We further detected markers of myelomonocytic cells, lymphoid cells, and HSPCs to confirm the cell components in R1, R2, and R3. The mRNA expression of GATA and RUNX1 was higher in the R3 fraction than in the R1 and R2 fractions (Supplemental Fig. 1A,B). The mRNA expression of PU.1 and EGR1 was higher in the R1 fraction compared with the R2 and R3 fractions (Supplemental Fig. 1C,D). The mRNA expression of PAX5 and GATA3 was higher in the R2 fraction compared with the R1 and R3 fractions (Supplemental Fig. 1E,F). These data support the hypothesis that myelomonocytic cells, lymphoid cells, and HSPCs are enriched in the R1, R2, and R3 fractions, respectively.

### HSPC egress after *L. anguillarum* infection

**Figure 2:**
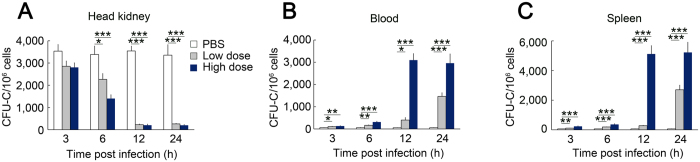
Haematopoietic progenitor cell egress after *L. anguillarum* challenge. *L. anguillarum* was injected i.p. into ayu in doses of 1.2 × 10^4^ or 1.2 × 10^5^ CFU/fish. The head kidney (**A**), blood (**B**), and spleen (**C**) were collected for further analysis. The number of CFU-Cs were analysed per millilitre of blood or per one million total head kidney cells or spleen cells. Values are shown as the means ± SEM; n = 5; *p < 0.05, **p < 0.01, ***p < 0.001.

To track the mobilisation of HSPCs after *L. anguillarum* challenge, we analysed the number of CFU-Cs in the head kidney, blood, and spleen using 0.1% PHA CM. We observed a significant decrease in the number of CFU-Cs in the head kidney (Fig. 2A). The number of CFU-Cs first decreased in the head kidney of *L. anguillarum*-infected ayu at 6 h post-infection (hpi) in both the low-dose and high-dose groups (Fig. 2A). The number of CFU-Cs in the high-dose group was only 5.3% of the PBS (control) group at 24 hpi (Fig. 2A). Moreover, the number of CFU-Cs dramatically increased in the blood and spleen after *L. anguillarum* infection (Fig. 2B,C). In the blood, the number of CFU-Cs first increased at 3 hpi in both the low-dose and high-dose groups (Fig. 2B). The number of CFU-Cs in the high-dose group increased 52.5 fold compared with the PBS group at 24 hpi (Fig. 2B). In the spleen, the number of CFU-Cs first increased at 3 hpi in both the low-dose and high-dose groups (Fig. 2C). The number of CFU-Cs in the high-dose group increased 83.5 fold compared with the PBS group at 24 hpi (Fig. 2C). These data suggest that the HSPCs in the head kidney mobilise into the blood after *L. anguillarum* infection.

### Norepinephrine (NE) mediates the effect of *L. anguillarum* infection-induced HSPC mobilization

TNFα, a pro-inflammatory cytokine, is up-regulated after infection^24^ and has been implicated in HSPC emergence^25^. Anti-TNF IgG was employed to investigate whether TNFα-mediated infection induced HSPC mobilisation. *In vitro* anti-TNF IgG treatment enhanced the number of CFU-Cs compared with isotype IgG (IsoIgG) treatment (Supplemental Fig. 2A). We further assayed the number of CFU-Cs in the head-kidney, blood, and spleen in ayu treated with anti-TNF IgG after *L. anguillarum* infection for 24 h. The number of CFU-Cs in the head kidney increased in the ayu injected with anti-TNF IgG compared with IsoIgG (Supplemental Fig. 2B). Furthermore, the number of CFU-Cs in the blood and spleen increased after anti-TNF IgG treatment (Supplemental Fig. 2C,D). These data do not support the hypothesis that TNF is the main mediator of HSPC mobilisation after infection in ayu.

The effect of ayu G-CSF on HSPC mobilisation after infection was further investigated. The cDNA sequence (GenBank accession number: JP740394) was identified as ayu G-CSF by multiple alignment and phylogenetic analysis (Supplemental Fig. 3). Moreover, we prokaryotically expressed recombinant G-CSF protein to prepare anti-G-CSF IgG (Supplemental Fig. 4A). Anti-G-CSF IgG treatment decreased the G-CSF protein levels in the plasma after *L. anguillarum* infection for 24 h (Supplemental Fig. 4B). We further assayed the number of CFU-Cs in the tissues of ayu treated with anti-G-CSF IgG after *L. anguillarum* infection for 24 h. The number of CFU-Cs in the head kidney, blood, and spleen did not change in ayu injected with anti-G-CSF IgG compared with IsoIgG after infection (Supplemental Fig. 4C–E). These data do not support the hypothesis that G-CSF is the main mediator of HSPC mobilisation after infection in ayu.

**Figure 3:**
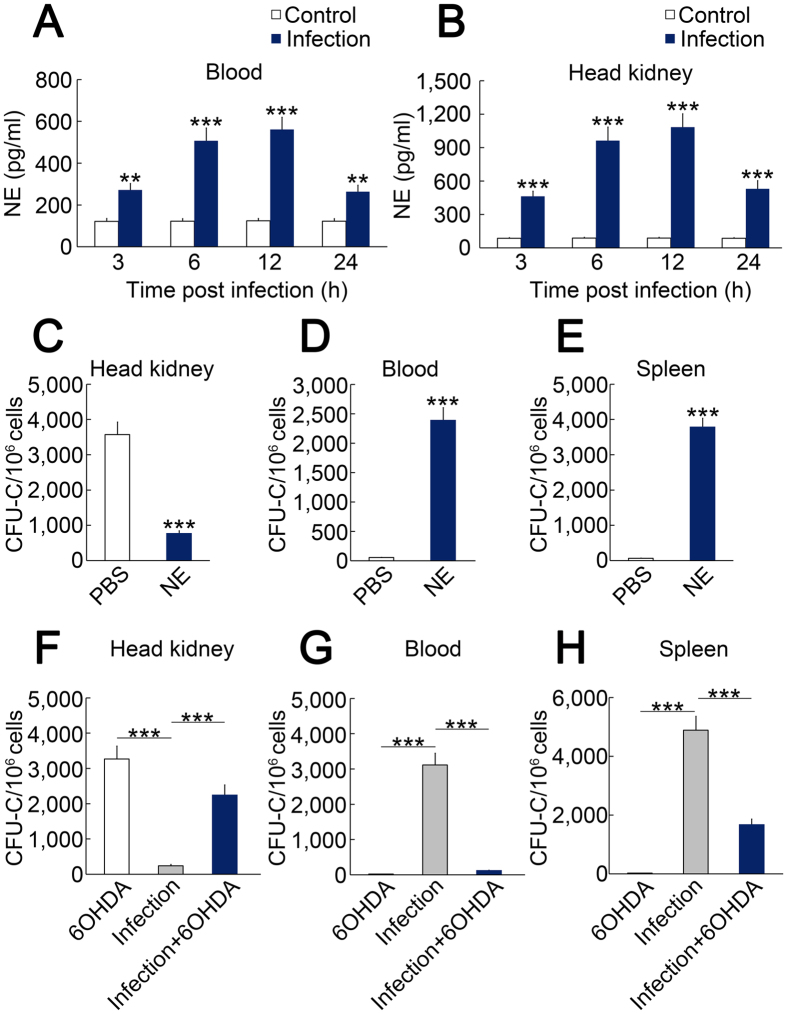
NE mediates infection-induced HSPC mobilisation. (**A,B**) NE levels were measured at different time points after infection. (**C–E**) The number of CFU-Cs in the head kidney, blood, and spleen after NE injection. (**F–H**) The number of CFU-Cs in the head kidney, blood, and spleen after infection with or without 6OHDA treatment. The data are expressed as the means ± SEM; *n* = 5; ***p < 0.001.

Infection leads to the up-regulation of NE in plasma^26^. Additionally, NE plays an important role in rapid HSPC mobilisation^27^. We further investigated whether NE mediates HSPC mobilisation after infection. NE levels were measured in the blood and head kidney at different time points after *L. anguillarum* infection. Blood NE levels were up-regulated at 3, 6, 12, and 24 hpi (Fig. 3A). The peak value of NE was 561.1 pg/ml at 12 hpi (Fig. 3A). The NE level in the head kidney was up-regulated at 3, 6, 12, and 24 hpi (Fig. 3B). The peak value of NE in the head kidney was 1083.1 pg/ml at 12 hpi (Fig. 3B).

Next, we investigated the role of NE in HSPC mobilisation. NE injection led to a decrease of CFU-Cs in the head kidney compared with PBS (Fig. 3C). The number of CFU-Cs in the blood and spleen increased 43.3-fold and 58.7-fold, respectively, after NE injection (Fig. 3D,E). Chemical sympathectomy was performed by treating ayu with 6-hydroxybutamine (6OHDA). 6OHDA increased the number of CFU-Cs in the head kidney after *L. anguillarum* infection (Fig. 3F). Furthermore, 6OHDA decreased the number of CFU-Cs in the blood and spleen in *L. anguillarum*-infected ayu (Fig. 3G,H). These results indicate that NE mediates HSPC mobilisation after *L. anguillarum* infection.

### HSPC enhance survival of irradiated ayu

**Figure 4:**
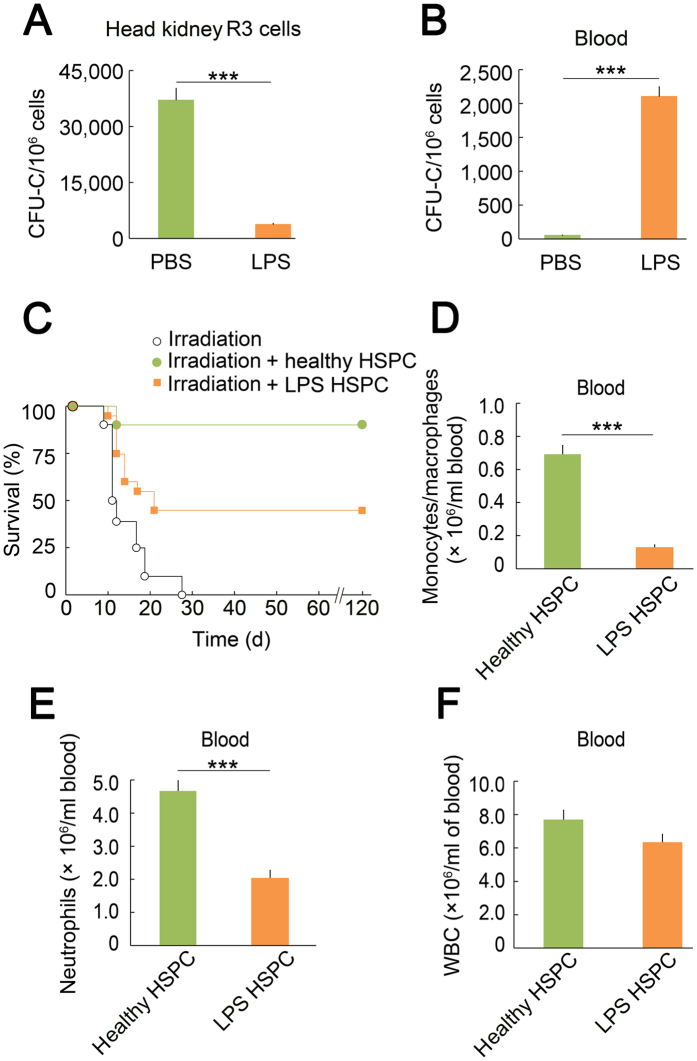
Effect of HSPCs on survival and myeloid cell numbers in irradiated ayu. *L. anguillarum* LPS (1 μg per ayu) was injected i.p. into ayu. The CFU-C numbers in the head kidney R3 cells (**A**) and blood (**B**) were further analysed. (**C**) Survival rate after the transplantation of HSPCs from healthy (1 × 10^4^ R3 cells) or *L. anguillarum* LPS HSPCs (1 × 10^5^ R3 cells), n = 20. HSPC transplantation affects the numbers of monocytes/macrophages (**D**), neutrophils (**E**), and total WBCs (**F**) in irradiated ayu. The data are expressed as the means ± SEM; n = 5; *p < 0.05, **p < 0.01, ***p < 0.001.

Because HSPCs from *L. anguillarum*-infected ayu may include live *L. anguillarum*, which affect the transplantation assay, we employed LPS prepared from *L. anguillarum* to mimic bacterial infection. After *L. anguillarum* LPS administration, we observed that the number of CFU-Cs in the head kidney R3 cells was 0.1-fold that of the head kidney in healthy ayu (Fig. 4A). Moreover, the number of CFU-Cs dramatically increased in the blood after *L. anguillarum* LPS administration (Fig. 4B). To study the differential roles of HSPCs from healthy ayu (healthy HSPC) or *L. anguillarum* LPS (LPS HSPC)-treated ayu on the haematopoietic system, we employed an HSPC transplantation method. Firstly, we measured the MHC I nucleotide sequence polymorphisms in the ayu. To exclude the presence of such polymorphisms, we sequenced the nucleotides of MHC I α2, which has been used in genotyping^28^. Complete nucleotide sequence homology was observed at the MHC I α2 domain in Zhemin No. 1 ayu. However, only 99.18% sequence identity was observed at the MHC I α2 domain in wild environmental ayu. Randomly selected Zhemin No. 1 ayu were divided into Group 1 and Group 2, while wild environmental ayu were classified as Group 3. Furthermore, we investigated lymphocyte-mediated cytotoxicity against allogeneic cells. Ayu peripheral blood lymphocytes (PBL) from Group 1 were able to lyse the erythrocytes from Group 3 (Supplemental Fig. 5). There was no erythrocyte lysis observed in Group 2 after adding PBL from Group 1.

We first confirmed the minimum lethal dose (MLD) of irradiation in the ayu. We administered graded doses of radiation to groups of 20 ayu in 5 Gy increments from 10 to 40 Gy. Animals receiving between 10 Gy and 20 Gy showed more than 20% survival (Supplemental Fig. 6A). The MLD, which we initially defined as less than 10% survival over 30 days, was 25 Gy (Supplemental Fig. 6A). Furthermore, we detected the CFU-Cs in the head kidney of irradiated ayu. The number of CFU-Cs in the head kidney from 25-Gy-irradiated ayu was only 0.87% of that from control ayu (Supplemental Fig. 6B).

*L. anguillarum* LPS HSPC transplantation led to a 45% survival rate in irradiated ayu (25 Gy), whereas healthy HSPC transplantation led to a 90% survival rate (Fig. 4C). The number of monocytes/macrophages, neutrophils, and WBCs in the blood were measured after HSPC administration in irradiated ayu. The number of monocytes/macrophages in LPS HSPC-treated ayu decreased compared with healthy HSPC-treated ayu (Fig. 4D). The number of neutrophils in LPS HSPC-treated ayu also decreased compared with healthy HSPC-treated ayu (Fig. 4E). However, no significant change was observed in total WBCs between healthy HSPC and LPS HSPC-treated ayu (Fig. 4F). Furthermore, the survival rate of ayu treated with *E. coli* LPS HSPCs was higher compared with *L. anguillarum* LPS HSPCs (Supplemental Fig. 7A). The number of monocytes/macrophages and neutrophils in the blood of ayu treated with *E. coli* LPS HSPCs increased compared with *L. anguillarum* LPS HSPCs (Supplemental Fig. 7B–D).

**Figure 5:**
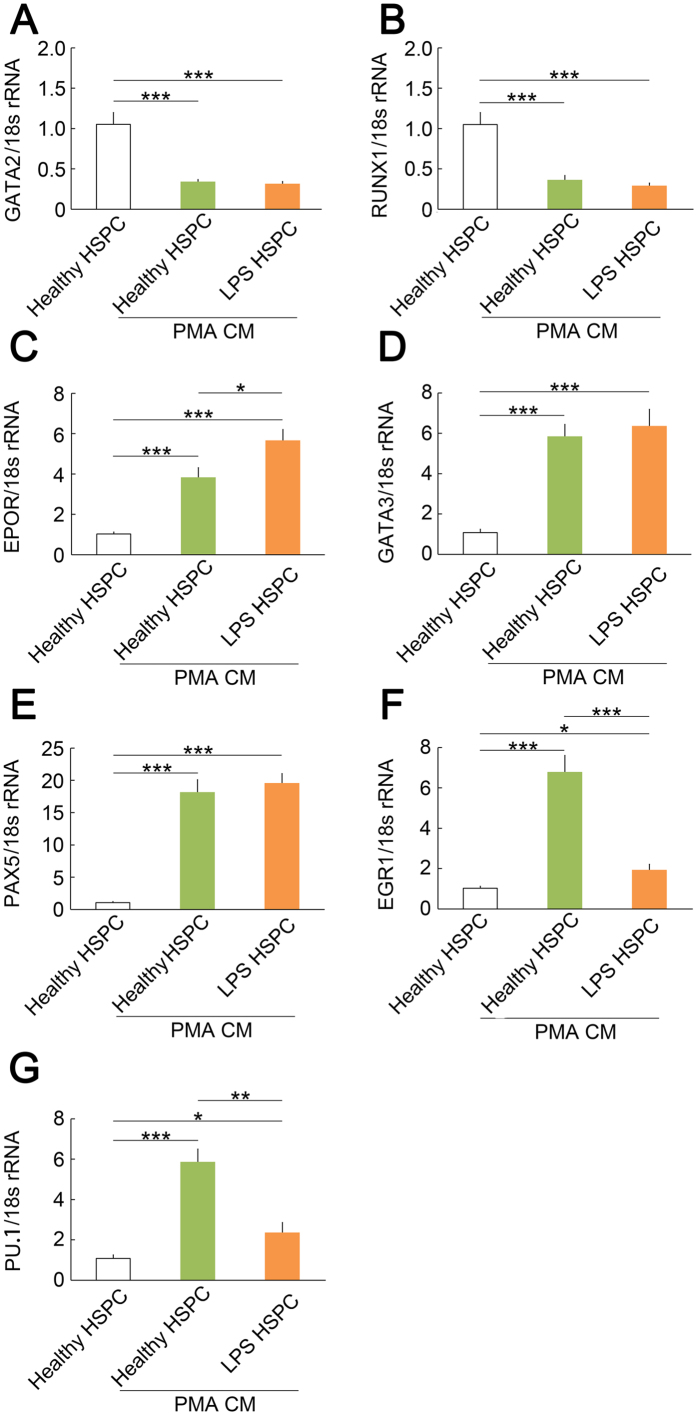
Quantitative expression analysis of ayu transcription factors in CFU-Cs. (**A**,**B**) mRNA expression of GATA2 and RUNX1 in CFU-Cs from *L. anguillarum* LPS HSPCs compared with healthy HSPCs. (**C**) mRNA expression of EPOR in CFU-Cs from *L. anguillarum* LPS HSPCs compared with healthy HSPCs. (**D,E**) mRNA expression of GATA3 and PAX5 in CFU-Cs from *L. anguillarum* LPS HSPCs compared with healthy HSPCs. (**F,G**) mRNA expression of EGR1 and PU.1 in CFU-Cs from *L. anguillarum* LPS HSPCs compared with healthy HSPCs. Cytokine gene transcripts were normalised to 18 s rRNA transcripts. The data are expressed as the means ± SEM; *n* = 6; *p < 0.05, **p < 0.01, ***p < 0.001.

To further characterise the differentiation capability of *L. anguillarum* LPS HSPCs, we examined the mRNA expression of genes involved in the development of various lineages of CFU-Cs. The mRNA levels of GATA2, RUNX1 (mainly expressed in HSPCs), EPOR (mainly expressed in erythroid cells), GATA3, PAX5 (mainly expressed in lymphocytes), EGR1, and PU.1 (mainly expressed in myeloid cells) were measured in healthy HSPCs treated with medium (control) or in healthy or LPS HSPCs treated with PHA CM for 48 h. Both PHA CM-treated healthy HSPCs and LPS HSPCs exhibited a significant down-regulation of GATA2 and RUNX1 compared with control (Fig. 5A,B). Both PHA CM-treated healthy HSPCs and LPS HSPCs exhibited a significant up-regulation of EPOR, GATA3, and PAX5 compared with control (Fig. 5C–E). PHA CM treated LPS HSPCs exhibited a significant up-regulation of EPOR compared with PHA CM-treated healthy HSPCs (Fig. 5C). However, the mRNA expressions of EGR1 and PU.1 were down-regulated in LPS HSPCs treated with PHA CM compared with healthy HSPCs treated with PHA CM (Fig. 5F,G). These data suggest that LPS HSPCs exhibit a weak differentiation capability toward myeloid cells *in vitro*.

### Effect of healthy HSPCs and LPS HSPCs on the outcome of *L. anguillarum*-infected ayu

**Figure 6:**
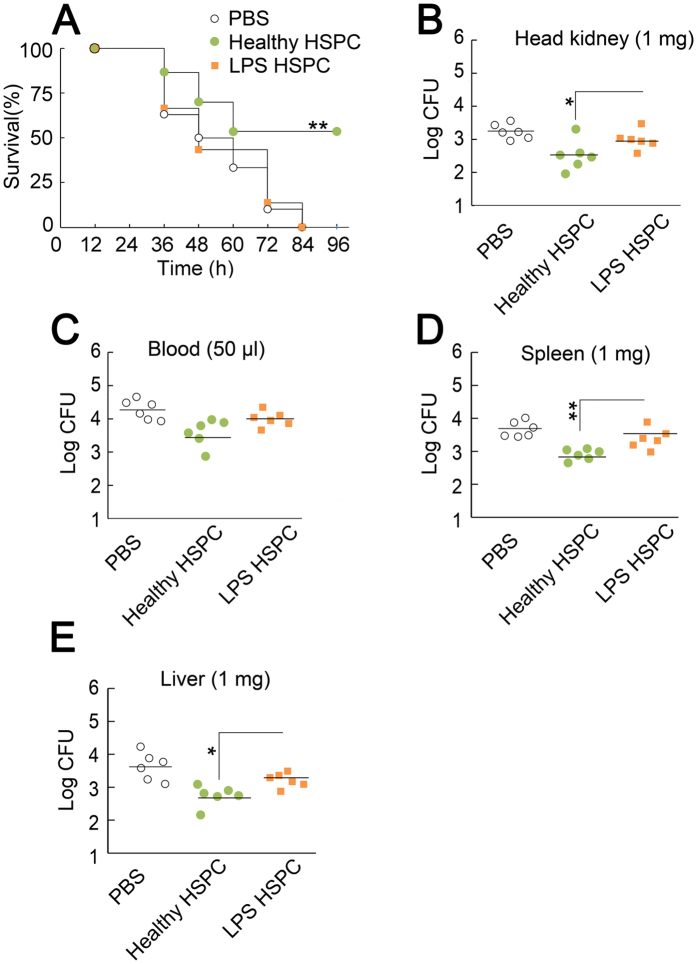
Effect of healthy or infected HSPCs on survival and bacterial burden in infected ayu. (**A**) Survival in ayu treated with healthy (1 × 10^4^ R3 cells) or *L. anguillarum* LPS HSPCs (1 × 10^5^ R3 cells). Fish were monitored for mortality every 12 h for 4 days. Experiments are representative of 30 fish per group. (**B**) The effect of healthy or LPS HSPCs on bacterial clearance in the ayu head kidney (**B**), blood (**C**), spleen (**D**), and liver (**E**) 24 h post-*L. anguillarum* infection. Tissue and blood homogenates were cultured on TCBS agar plates. Colony numbers were normalised to tissue weight (for tissue) and volume (for blood). n = 5 in each group; **p < 0.01, ***p < 0.001.

Healthy HSPCs or *L. anguillarum* LPS HSPCs were infused into ayu that were simultaneously infected with *L. anguillarum. L. anguillarum* infection resulted in a dramatic reduction in the survival rate during the 96 h after PBS treatment (Fig. 6A). Healthy HSPCs rescued the survival rate of *L. anguillarum-*infected ayu, while LPS HSPCs did not affect survival (Fig. 6A). The survival rate of healthy HSPC-infused ayu was 53.3%.

Next, we employed the plate count method to assay the bacterial burden in tissues. *L. anguillarum* was not detectable in the head kidney, blood, spleen, or liver of healthy fish. The number of colony-forming units (CFU) per mg of tissue or per 50 μl of blood in *L. anguillarum-*infected ayu is shown in Fig. 6B–E. The bacterial burden in the head kidney from the *L. anguillarum* LPS HSPC group was 1.2 × 10^3^ CFU per mg, whereas that from the healthy HSPCs group was 5.2 × 10^2^ CFU per mg (Fig. 6B). The bacterial burden in the blood from the LPS HSPC group was 1.1 × 10^4^ CFU per 50 μl, whereas that from the healthy HSPC group was 5.0 × 10^3^ CFU per 50 μl (Fig. 6C). The bacterial burden in the spleen from the LPS HSPC group was 3.0 × 10^3^ CFU per mg, whereas that from the healthy HSPC group was 8.2 × 10^2^ CFU per mg (Fig. 6D). The bacterial burden in the liver from the LPS HSPC group was 1.7 × 10^3^ CFU per mg, whereas that from the healthy HSPC group was 6.5 × 10^2^ CFU per mg (Fig. 6E).

**Figure 7:**
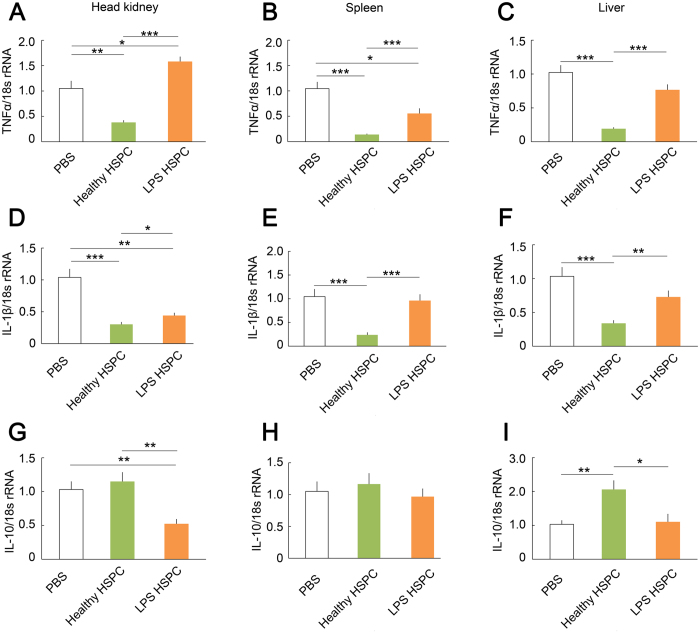
RT-qPCR analysis of cytokine gene transcripts in infected ayu after HSPC infusion. TNFα, IL-1β, and IL-10 transcripts at different time points were detected following *L. anguillarum* infection. Fish were sacrificed at 24 hpi. Cytokine gene transcripts were normalised to 18 s rRNA transcripts. The data are expressed as the means ± SEM; n = 6; *p < 0.05, **p < 0.01, ***p < 0.001.

RT-qPCR was performed to analyse TNFα, IL-1β, and IL-10 transcripts in the head kidney, spleen, and liver of healthy HSPCs or *L. anguillarum* LPS HSPCs-infused ayu that were infected with *L. anguillarum*. Healthy or LPS HSPC infusion led to a significant alteration of cytokine expression. After healthy HSPC infusion into infected ayu, the expression of TNFα mRNA was down-regulated in the head-kidney, spleen, and liver compared with PBS infusion, whereas the expression of TNFα mRNA was up-regulated in LPS HSPC-infused ayu compared with healthy HSPC-infused ayu (Fig. 7A–C). The expression of IL-1β mRNA was down-regulated in the head kidney, spleen, and liver of ayu infused with healthy HSPCs compared with ayu receiving PBS infusion, whereas the expression of IL-1β mRNA was up-regulated in the head kidney, spleen, and liver of ayu infused with LPS HSPCs compared with ayu infused with healthy HSPCs (Fig. 7D–F). The expression of IL-10 mRNA was similar in the spleen of ayu infused with healthy HSPCs compared with ayu infused with LPS HSPCs (Fig. 7H). The expression of IL-10 mRNA was down-regulated in the head-kidney and liver of ayu infused with LPS HSPCs compared with ayu receiving healthy HSPCs (Fig. 7G,I).

### *L. anguillarum* infection suppresses HSPC expansion *in vitro*

**Figure 8:**
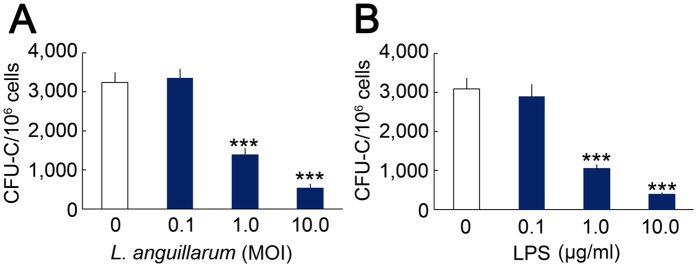
*L. anguillarum* and its LPS inhibit CFU-Cs. (**A**) Effect of *L. anguillarum* on CFU-Cs. (**B**) Effect of *L. anguillarum* LPS on CFU-Cs. The data are expressed as means ± SEM; n = 5; ***p < 0.001.

Because healthy HSPCs can rescue the immune system of ayu infected with *L. anguillarum*, whereas LPS HSPCs cannot, we investigated the effect of *L. anguillarum* and its LPS on HSPC expansion *in vitro. L. anguillarum* infection did not change the number of CFU-Cs at a multiplicity of infection (MOI) of 0.1, whereas it decreased the number of CFU-Cs at MOIs of 1.0 and 10 (Fig. 8A). *L. anguillarum* LPS did not change the number of CFU-Cs at a concentration of 0.1 μg/ml, whereas it decreased the number of CFU-Cs at concentrations of 1.0 and 10 μg/ml (Fig. 8B).

## Discussion

HSPCs can proliferate and mobilise into the blood in response to infection in mammals^29^. However, the process and function of HSPCs after infection in teleosts are less well known. We found that the PHA CM stimulated the formation of CFU-Cs, which represents HSPC activity. Furthermore, after infection, the number of CFU-Cs increased in the blood and spleen but decreased in the head kidney, suggesting that infection can induce HSPC mobilisation from the head kidney into the blood. We also found that *L. anguillarum*-induced HSPC mobilisation into the blood was mediated by NE but not TNFα. Furthermore, HSPC-enriched cells isolated by flow cytometry could rescue the immune system of irradiated ayu. Ayu engrafted with HSPCs from *L. anguillarum-*infected ayu showed lower numbers of monocytes/macrophages and neutrophils but similar numbers of total WBCs compared with ayu engrafted with healthy HSPCs. Hence, *L. anguillarum* infection reduced the capability of HSPCs to differentiate into myeloid cells. This is the first time that HSPC homeostasis and function have been elucidated in aquacultured fish following infection.

It is impossible to precisely define HSPCs in ayu only by FSC and SSC. According to the RT-qPCR data, HSPC marker genes were more highly expressed in the R3 fraction compared with the R1 and R2 fractions. Furthermore, CFU-Cs in the R3 fraction were higher compared with the R1 and R2 fractions, suggesting that the R3 fraction is an HSPC-enriched cell subset. We also found that 3.7% of the cells in the R3 fraction were HSPCs. Additionally, we detected lymphocyte marker genes in R3. Hence, the R3 fraction may also include lymphocytes, as was found in zebrafish^14^. Separation of lymphocytes and HSPCs in teleosts only by light-scatter characteristics is challenging. Our investigation provides a basis for the isolation of an HSPC-enriched cell subset to study gene expression and functional analysis. However, further investigation is needed to purify HSPCs in the ayu in future studies.

We established a new method to assay the CFU-Cs of HSPC homeostasis in ayu by adding PHA CM. In mammals, conditioned media of PHA-stimulated PBMC have been used to assay HSPCs^30^. Alternatively, protein markers on the cell surface are often used to assay HSPCs. In mice, lineage^−^c-kit^+^Sca-1^+^ (LSK) cells are thought to be HSPC-enriched cells^31^. Furthermore, human HSPCs are isolated based on CD34, CD38, and CD90 expression^32^. However, it is difficult to find this many protein markers in aquacultured fish. In zebrafish, a method has been established to assay the number of HSPCs by a GFP transgene^14^,^33^. This is a good method to qualitatively measure the number of HSPCs. However, it cannot be used in non-model organism research. In goldfish, model systems have been established to measure myelopoiesis^34^, granulopoiesis^35^, erythropoiesis^15^,^16^, and thrombopoiesis^36^. *In vivo* transplantation to test the repopulation activity of donor cells has been the gold standard for the characterisation of HSPCs. In ginbuna carp, a unique transplantation model system was established for detecting HSPCs and HPCs using clonal ginbuna crucian carp (*Carassius auratus langsdorfii*, S3n strain) and ginbuna-goldfish (*Carassius auratus*, S3n strain) hybrids^37^. Although haematopoiesis in teleosts has been investigated, HSPC homeostasis after infection in teleosts is less understood. Because HSPCs play an important role upon mobilisation into the blood after infection^38^, we developed a useful method to detect HSPC homeostasis in aquacultured fish by adding PHA CM to the HSPC culture.

Using this CFU-C assay, we further found that *L. anguillarum* infection led to HSPC mobilisation into the blood. Although HSPCs are frequently dormant, they are thought to participate directly in the primary response to infection in mammals. *E. coli* stimulates the expansion and mobilisation of LSK cells^39^. Furthermore, the LSK cell compartment is stimulated in models of other bacterial and viral infections^40^,^41^. Therefore, it seems that HSPC mobilisation into the blood is a common pathway in the immune response after pathogen infection in fish and mammals. The spleen is a secondary haematopoietic organ in fish^42^. We found that the number of CFU-Cs was dramatically up-regulated after infection. Our results support the concept that the haematopoietic function of the spleen is enhanced after infection. However, it remains unclear what factor mediates the effect of *L. anguillarum* infection on HSPC mobilisation. Several pathways for the effect of infections on HSPCs may include the following: (1) pro-inflammatory cytokines may signal to HSPCs; (2) pathogen-derived products may be sensed by HSPCs; and (3) changes in the niche may occur in response to infection^6^. TNFα is the primary pro-inflammatory cytokine after infection in teleosts^43^, and TNFα regulates the emergence of HSPCs in zebrafish^25^. In the present study, anti-TNF and anti-G-CSF IgGs did not affect HSPC mobilisation in infected ayu. The data do not support the hypothesis that TNF and G-CSF mainly mediate HSPC mobilisation after infection in ayu. In the present study, we found that NE mediated the effect of *L. anguillarum* infection on HSPC mobilisation. NE acts as a modulator in the immune system of animals^44^,^45^. In mammals, NE injection for 1 hour was found to increase the number of CFU-Cs by approximately 2-fold by affecting plasma levels of stromal cell-derived factor-1^27^. In ayu, NE injection increased the number of CFU-Cs in the blood by 43.3-fold. NE is the primary neurotransmitter released from the sympathetic nervous system. We further employed 6OHDA to disrupt the sympathetic nervous system, and 6OHDA treatment diminished HSPC mobilisation after infection. Because 6OHDA damages both central and peripheral dopaminergic and noradrenergic neurons, further investigation is necessary to illustrate the function of the sympathetic nervous system on HSPC mobilisation. These data generally support the notion that NE is the main mediator of HSPC mobilisation after infection in ayu.

Furthermore, we found that *L. anguillarum* LPS HSPCs functioned differently compared with healthy HSPCs. LPS HSPCs were less able to rescue the immune system of irradiated ayu and produced fewer myeloid cells. However, the HSPCs from *E. coli* LPS-treated ayu still maintained normal reconstitution potential. In a polymicrobial sepsis model using caecal ligation and puncture, HSPCs from septic mice retained sufficient reconstitution potential in transplant assays to allow survival of lethally irradiated recipients^46^. However, *Pseudomonas aeruginosa*-infected mice displayed reduced myeloid differentiation, and HSPCs from infected animals did not engraft well after transplantation into lethally irradiated recipient mice^38^. Hence, the deficiency of HSPCs from infected animals may be attributed to infection and pathogenesis. *L. anguillarum* is the main pathogen of ayu^47^. In our previous study, we demonstrated an important role for monocytes/macrophages against *L. anguillarum*^47^. In the present study, we also found that LPS HSPCs exhibited fewer numbers of monocytes/macrophages and neutrophils after transplantation. The CFU-Cs from LPS HSPCs showed down-regulated mRNA expression of EGR1 and PU.1, which are markers of myeloid cells. Therefore, the high mortality due to *L. anguillarum* infection in ayu may result from the reduced differentiation of myeloid cells, including neutrophils and monocytes/macrophages. We also found that the CFU-Cs from LPS HSPCs showed up-regulated mRNA expression of EPOR, which is a marker of erythrocytes. These data suggest that LPS HSPCs may enhance erythrocyte differentiation, which is consistent with previous findings showing that infection increases erythrocyte numbers in other fish^48^. Additionally, it is noteworthy that *L. anguillarum* is the pathogen of rainbow trout^49^ and seabream^50^. Monocytes/macrophages play an important role against infection^51^,^52^,^53^. Therefore, it is of interest to further investigate whether *L. anguillarum* infection has a similar effect on myeloid cell differentiation in teleosts other than ayu.

In zebrafish, a GFP-labelled strain has been established to observe the homing, differentiation, and proliferation of HSPCs^33^. In the carp, a triploid strain has been established to label transplanted cells^37^. A label technology is not currently available to distinguish transplanted cells from host cells in ayu. Further investigation is needed to develop a sex marker or triploid strain in ayu before studying the homing, differentiation, or proliferation of HSPCs.

In summary, our study reveals that *L. anguillarum* infection induces HSPC mobilisation, which is mediated by NE. The HSPCs from *L. anguillarum* LPS-treated ayu display deficient reconstitution potential and myeloid differentiation compared with HSPCs from healthy ayu. Our study documents HSPC homeostasis and function after infection in ayu and suggests that HSPCs play a previously unknown role in the response to infection in teleosts.

## Methods

### Fish

Ayu (Zhemin No. 1, weighing 40 ± 5 g each) were used in all experiments. This strain has undergone successive seven-generation of mass selection with repeat breeding. The wild environmental ayu were obtained from Fuxi river in Ningbo City, China. Healthy fish were kept in a 100-liter tank at 20 °C with regular feeding. The fish were cultured under laboratory conditions for two weeks before the experiments were performed. All animal work in this paper was conducted according to relevant national and international guidelines. All animal care and experimental procedures were approved by the Committee on Animal Care and Use and the Committee on the Ethics of Animal Experiments of Ningbo University.

Ayu were injected intraperitoneally (i.p.) with *L. anguillarum* at two different doses, 1.2 × 10^4^ (low dose), and 1.2 × 10^5^ (high dose) CFU/fish. After administration, the colony-forming unit-culture (CFU-C) counts in the head kidney, blood, and spleen were determined.

### Reagents

Anti-TNF IgG, prepared as previously described^54^, was injected i.p. at a dose of 300 μg/kg body weight. 6OHDA (Sigma, USA) was injected i.p. at a dose of 100 mg/kg body weight. NE (Sigma) was injected i.p. at a dose of 5 mg/kg body weight.

LPS was prepared from *L. anguillarum* using the proteinase K method. Cells were washed and resuspended in saline. A cell pellet obtained from 1.5 ml of the bacterial suspension was resuspended in the lysis buffer (5% 2-mercaptoethanol, 10% glycerol, 10% (w/v) sodium dodecyl sulfate, in 0.5 M Tris-HC1, pH 6.8) and heated at 100 °C for 30 min. An aliquot of 20 μl of 0.25% w/v proteinase K (Sigma) in sample buffer was added to the sample and the mixture was incubated at 60 °C for 1 h. The resulting LPS samples were stored at −20 °C. LPS was administered i.p. at a dose of 1 μg per ayu. Ayu was killed 24 h after LPS administration.

Based on the previously determined G-CSF sequence (GenBank accession number: JP740394), a primer pair was designed that would amplify the ORF and which included restriction sites for *Bam*H I and *Xho* I (Table 1). The amplicon was subsequently cloned into pET-28a. Pfu DNA Polymerase (Fermentas) was used for gene amplification according to the manufacturer’s protocols. Prokaryotic over-expression of the protein was used established protocols^24^. The His-tagged recombinant proteins were purified using a nickel-nitrilotriacetic acid column (QIAGEN) and analyzed by SDS-PAGE. The recombinant protein was used to immunize mice to produce antisera. Protein A HP SpinTrap columns (GE healthcare, New Jersey, USA) were used to purify anti-G-CSF IgG from prepared antisera. The anti-G-CSF IgG was injected i.p. at a dose of 300 μg/kg body weight.

### Enzyme-linked immunosorbent assay (ELISA)

An ELISA was developed using the anti-G-CSF IgG. Micro plates (Nunc ImmunoPlates) were coated using ayu plasma overnight. Plates were precoated using Poly-L-Lysine (Sigma Aldrich) to increase protein binding. Blocking of unbound binding sites was performed using 5% teleost gelatin (Sigma Aldrich). Anti-G-CSF IgG was added to each well, incubated for 1 h and washed three times. The secondary conjugated antibody was added to each well for 1 h and washed three times. Finally, alkaline phosphatase yellow liquid substrate system for ELISA (Sigma Aldrich) was used and optical density read at 405 nm.

### *In vitro* CFU-C assays

The assay for CFU-Cs was carried out as described previously^55^. Briefly, we plated mononuclear cells (MNCs) in 2.5 ml methylcellulose medium supplemented with 10% conditioned medium obtained from PBMC in the presence of phytohemagglutinin (Sigma) for 7 days. The numbers of NMCs were 2 × 10^5^/plate from head kidney, 1 × 10^6^/plate from blood, or 1 × 10^6^/plate from spleen. After 7 days of culture, the number of colonies per dish was counted.

### Flow cytometry

Haematopoietic cells isolated from ayu were processed as described previously in zebrafish^14^. Head kidney was isolated, washed and resuspended in ice cold PBS plus 5% FBS, and was passed through a filter with a 40-μm pore size. MNCs were isolated by centrifugation at 400 × g for 25 min on Ficoll (GE Healthcare). Flow cytometry analysis was based on FSC and SSC on the flow cytometry (Gallios, Beckman Coulter, Miami, USA). HSPC-enriched head kidney cells were also sorted using the MoFlo XDP cell sorter (Beckman Coulter), defined as R3 cells.

### Real-time quantitative PCR (RT-qPCR)

The RT-qPCR was carried out as reported previously^24^. Total RNA was extracted and purified from tissues or cells using RNAiso reagents (TaKaRa, Dalian, China). After deoxyribonuclease I treatment, cDNA was synthesised using reverse transcription M-MLV (TaKaRa). The primers used are listed in Table 1. The RT-qPCR protocol used was: 94 °C for 5 min, 40 cycles of 94 °C for 30 s, 60 °C for 30 s, and 72 °C for 30 s, in a RT-Cycler^TM^ real-time fluorescence quantitative PCR thermo-cycler (CapitalBio, Beijing, China). The mRNA expression of genes was normalised against that of 18s rRNA, and the quantitative difference in expression between different samples was calculated using the 2^−ΔΔCt^ method^56^.

### Irradiation

The recipient ayu were placed in an acrylic container (28 cm diameter, 8 cm depth) between two opposing ^137^Cs γ-ray sources (Gamma cell 40; Nordion International, Kanata, Ontario, Canada). The ayu were then exposed to various fractionated total-body irradiation at a rate of 1.02 Gy/min before transplantation.

### Amplification of ayu MHC I α2 domain and DNA sequencing

The cDNA sequence of ayu (Accession No. KX228907) was identified as ayu MHC I by multiple alignment and phylogenetic analysis. Ayu MHC I is identified by using rainbow trout MHC I amino acid sequence (AAQ18009) to screen a transcriptomic database of ayu head kidney-derived monocytes/macrophages. The sequence of α2 domain of ayu MHC I, which is important for interaction with antigenic peptide^28^, was used to measure the polymorphism of MHC I. Genomic DNA was extracted from head kidney of fish with a DNA Extraction Kit (TaKaRa, Dalian, China), dissolved in TE buffer, and stored at −80 °C before use. LA Taq DNA polymerase (TaKaRa). The sequences of ayu MHC I α2 domain were amplified by PCR with primers (Table 1). Thermal cycling conditions for PCR were as follows: 94 °C for 10 min, 30 cycles of 94 °C for 30 s, 58 °C for 30 s, and 72 °C for 1 min, and a final extension at 72 °C for 10 min. The PCR product was cloned into the pMD19-T Simple Vector (TaKaRa) and sequenced (Invitrogen, Shanghai, China). For each fragment, the complete sequences from at least three clones were determined to exclude PCR mistakes.

### Erythrocyte lysis by lymphocytes

We employed erythrocyte lysis assay to investigate the allograft rejection as previously described^57^. The PBMC were separated using Ficoll-Hypaque PREMIUM (1.077 g/ml) (GE Healthcare) in combination with centrifugation according to the manufacturer’s instructions. Ayu anti-CSF1R antibody was prepared in our lab^58^. PBMC were incubated with primary ayu anti-CSF1R antibodies for 30 min at 4 °C. Cells were then incubated with goat-anti-mouse-Ig microbeads (Miltenyi Biotec, Germany) after washing twice. Unlabeled cells flowing through the column were collected as ayu PBL.

Ayu erythrocytes were prepared as target cells^57^. To quantify the killing activity of alloreactive PBL, a hemoglobin release assay was carried out^57^. In this assay effector cells are co-cultured with erythrocyte targets and hemoglobin released from killed erythrocytes into the supernatant is measured colorimetrically using 3,3′,5,5′-tetramethylbenzidine (TMB). The percentage of specific cytotoxicity was calculated using the following formula: % Specific cytotoxicity = [(EXP–ESR)–TSR]/[(TMR–VCC) - TSR] × 100%. EXP: experimental wells were filled with varying numbers of ayu PBL and a constant number of target erythrocytes, ESR: effector cell spontaneous release, TSR: target cell spontaneous release, TMR: target cell maximum release, VCC: volume correction control.

### Transplantation

Cells for transplantation were obtained from ayu head kidney. Single cell suspensions of head kidneys were prepared using a 100-μm wire mesh, layered using a Ficoll gradient (GE Healthcare), and subsequently centrifuged at 400 × g for 25 min. The leukocyte fraction in the Ficoll-medium interface was collected and resuspended in PBS. HSPC-enriched head kidney cells were sorted using the MoFlo XDP cell sorter (Beckman Coulter) and defined as R3 cells. 1.0 × 10^4^ R3 cells from healthy ayu and 1.0 × 10^5^ R3 cells from infected ayu at 24 hpi were injected into ayu via caudal vessels.

### Measurement of plasma levels of NE

After infection, blood samples were simultaneously collected into tubes containing ethylene glycocol tetraacetic acid and reduced glutathione to prevent blood clotting and NE degradation. Plasma was immediately separated and stored at −70 °C until assayed. Plasma NE was determined using high-pressure liquid chromatography (HPLC) with electrochemical detection^59^.

### Survival assay and bacterial burden

For survival assay, morbidity was monitored after infection and HSPC transplantation. Morbidity was monitored for 96 h after challenge, and the results were recorded every 12 h.

Bacterial burden was measured as colony-forming units per mg tissue or per 50 μl of blood as described previously^60^. Briefly, head kidney, blood, spleen, and liver were harvested aseptically from ayu at 24 hpi. The tissues from each ayu were weighed and homogenized in 1 ml of sterile PBS. Homogenates and blood were diluted serially in sterile PBS (pH 7.2) and then plated onto separate Thiosulfate Citrate Bile Salts (TCBS) agar plates for 18 h at 28 °C.

### Cell count

For monocytes/macrophages count, the anti-CSF1R IgG was labelled to the cells according to the method reported previously^58^. Blood was collected and the cells were layered on Ficoll. After 25 min centrifugation, the leukocyte fraction in the Ficoll-medium interface was collected and incubated with mouse anti-CSF1R-Ex IgG (250 mg/ml) for 0.5 h at 4 °C. As a control, IsoIgG (250 mg/ml) were added. After washing, cells were incubated with fluorescein isothiocyanate labelled anti-mouse IgG for 0.5 h at 4 °C. Finally, the washed cells were counted by flow cytometry using the Gallios Flow Cytometer (Beckman Coulter, Miami, FL, USA). For neutrophil counts, the packed cells below the Ficoll (containing erythrocytes and granulocytes) were exposed to two rounds of hypotonic lysis treatment with distilled water for 30 s each. The granulocyte suspension obtained was stained with Giemsa. The number of neutrophils was determined with a haemocytometer.

### Statistical analysis

Results are presented as mean ± standard error of the mean (SEM). We analysed the survival curves using the Kaplan-Meier method with SPSS (version 13.0, Chicago, IL, USA). Other data were subjected to one-way analysis of variance (ANOVA). *P*-values < 0.05 were considered statistically significant.

## Additional Information

**How to cite this article**: Lu, X.-J. *et al*. Mobilisation and dysfunction of haematopoietic stem/progenitor cells after *Listonella anguillarum* infection in ayu, *Plecoglossus altivelis. Sci. Rep.* **6**, 28082; doi: 10.1038/srep28082 (2016).

## Supplementary Material

Supplementary Information

## Figures and Tables

**Table 1:** Oligonucleotide primers used in this work.

**Primers**	**Nucleotide sequence (5**′**–3**′)	**Gene**	**Accession number**
PaG-CSFF	GGGATCCGCCCCTCTTGAGGGATATT	G-CSF	JP740394
PaG-CSFR	GCTCGAGCTACATAGCGTCCAATGCC		
PaGATA2F	TGTGCTAACTGCCAGACGAC	GATA2	KU833214
PaGATA2R	GGCTCTTTTTGGACTTGCTG		
PaRUNX1F	CATCCACCACCCTCTCATCT	RUNX1	KU833216
PaRUNX1R	GTCCGTTCTCACCAGCTCTC		
PaEPORF	AGCAGTCCTGGATCCTCTCA	EPOR	KU833212
PaEPORR	TCACCGCCATAAACTGTGAA		
PaGATA3F	GTGGCTTGAAGGAAGCAAAG	GATA3	KU833213
PaGATA3R	CGGGTCTGGAGACACATCTT		
PaPAX5F	CGTGTGTGTGACAACGACAG	PAX5	KU833217
PaPAX5R	CGCTGATGGAGTAGGAGGAG		
PaEGR1F	AGCCCAACCCCATCTACTCT	EGR1	KU833211
PaEGR1R	AAGCTGGAACTGCACGTCTT		
PaPU.1F	GAGCTCAGACGAGGACGAAC	PU.1	KU833215
PaPU.1R	CCGTTCCTCAACAGGTCAAT		
PaTNFαF	ACATGGGAGCTGTGTTCCTC	TNFα	JP740414
PaTNFαR	GCAAACACACCGAAAAAGGT		
PaIL-1βF	TACCGGTTGGTACATCAGCA	IL-1β	HF543937
PaIL-1βR	TGACGGTAAAGTTGGTGCAA		
PaIL-10F	TGCTGGTGGTGCTGTTTATGTGT	IL-10	JP758157
PaIL-10R	AAGGAGCAGCAGCGGTCAGAA		
PaMHCIαF	GGTATGCACATATTTCAGTACA	MHC I	KX228907
PaMHCIαR	TTTCTTCTTTAGAGTGCTTTTCC		
Pa18S rRNAF	GAATGTCTGCCCTATCAACT	18S rRNA	FN646593
Pa18S rRNAR	GATGTGGTAGCCGTTTCT		
