# Supplementary material for: Mobilisation and dysfunction of haematopoietic stem/progenitor cells after Listonella anguillarum infection in ayu, Plecoglossus altivelis
Source: Sci Rep. 2016 Jun 16;6:28082. doi: 10.1038/srep28082 (PMC4910102; doi:10.1038/srep28082)
Supplement: Supplementary Information [file srep28082-s1.doc]

**Supplementary Information**

**Mobilisation and dysfunction of haematopoietic stem/progenitor cells after *Listonella anguillarum* infection in ayu, *Plecoglossus altivelis***

Xin-Jiang Lu^1^, Qiang Chen^1,2^, Ye-Jing Rong^1^, Jiong Chen^1,2,*^

^1^Laboratory of Biochemistry and Molecular Biology, School of Marine Sciences, Ningbo University, Ningbo 315211, China. ^2^Collaborative Innovation Center for Zhejiang Marine High-efficiency and Healthy Aquaculture, Ningbo University, Ningbo 315211, China. ^*^Corresponding author. Tel: +86 574 87609571; Fax: +86 574 87600167; E-mail address: jchen1975@163.com (J. Chen)


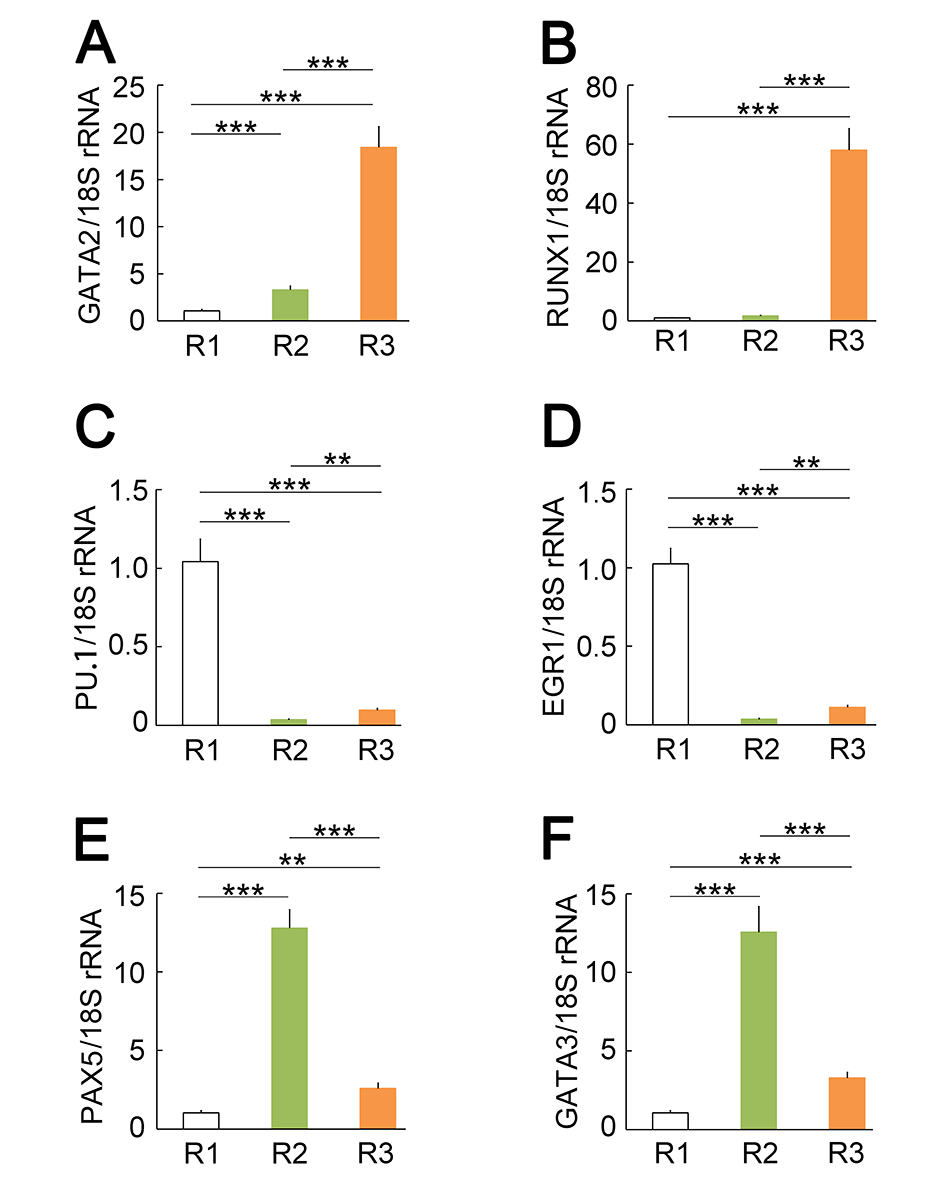


**Supplemental Figure 1. Quantitative expression analysis of ayu transcription factors in CFU-Cs.** (A, B) mRNA expression of GATA2 and RUNX1 in R1, R2, and R3 fractions. (C, D) mRNA expression of PU.1 and EGR1 in R1, R2, and R3 fractions. (E, F) mRNA expression of PAX5 and GATA3 in R1, R2, and R3 fractions. Cytokine gene transcripts were normalised to 18S rRNA transcript. Data are expressed as the mean ± SEM. *n* = 6. **p*<0.05, ***p*<0.01, ****p*<0.001.


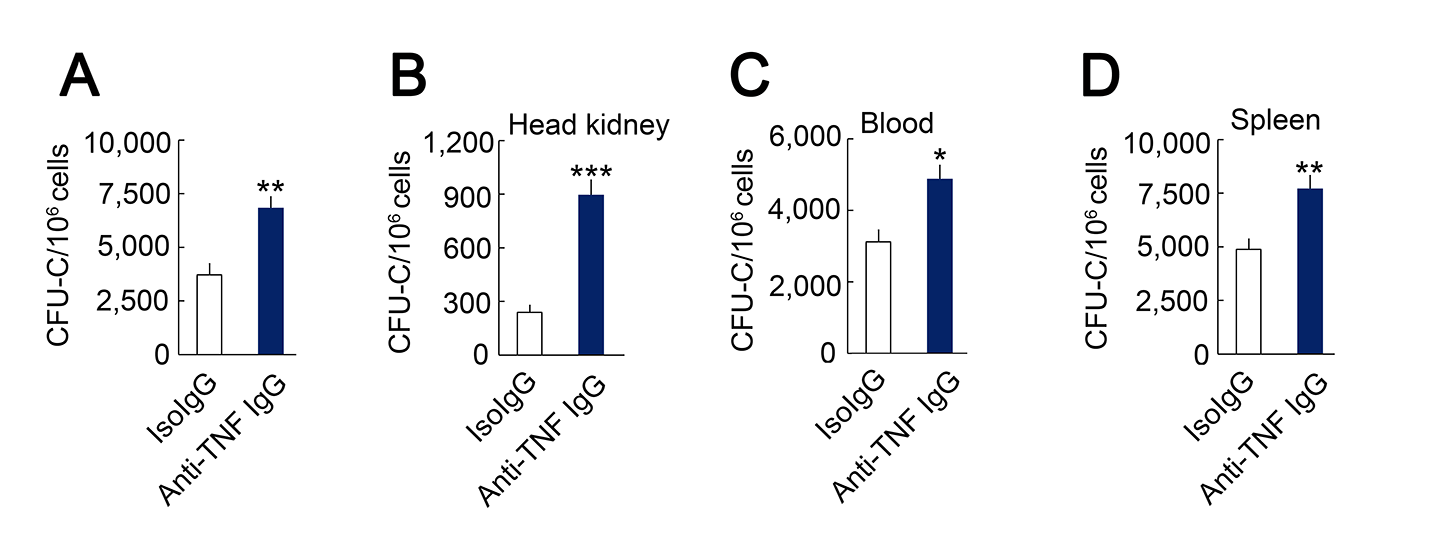


**Supplemental Figure 2. Effect of anti-TNF IgG on the CFU-Cs.** (A) The number of CFU-Cs after anti-TNF IgG treatment *in vitro*. (B-D) The number of CFU-Cs in cells from the head kidney, blood, and spleen of ayu treated with anti-TNF IgG after *L. anguillarum* infection for 24 h. Data are expressed as mean ± SEM. n = 5. **p*<0.05, ***p*<0.01, ****p*<0.001.


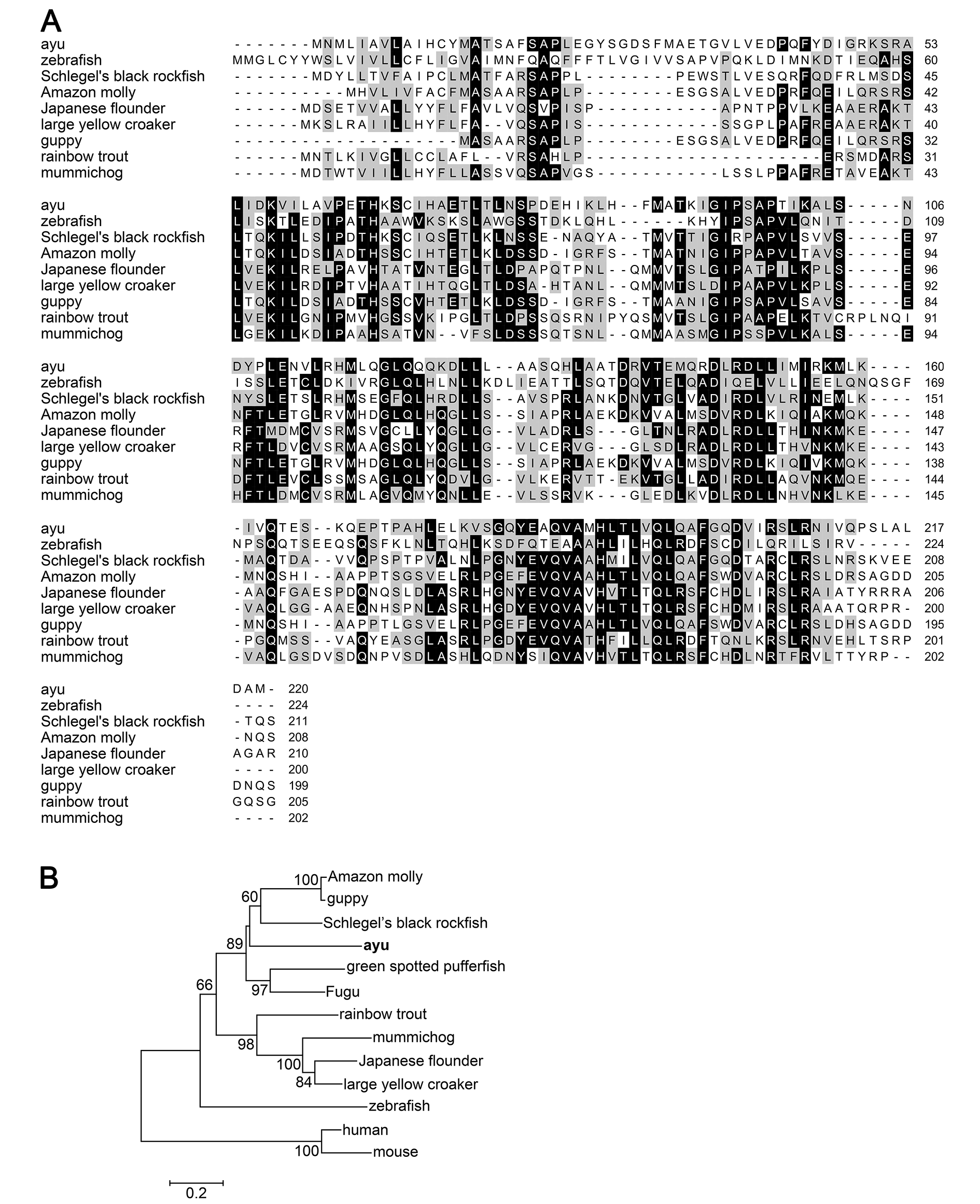


**Supplemental Figure 3. Multiple alignment and phylogenetic analysis of the amino acid sequences of ayu G-CSF and other closely related animal G-CSFs.** (A) Multiple alignment of fish G-CSFs. Threshold for shading was > 60% of similarity. Similar residues are shadowed gray and identical residues are shadowed black. (B) Phylogenetic (Neighbor-joining) analysis of amino acid sequences of G-CSF using the MEGA5.0 program. The values at the forks indicate the percentage of trees in which this grouping occurred after bootstrapping (1000 replicates; shown only when >60%). The accession numbers of G-CSF sequences are JP740394 for ayu (*Plecoglossus altivelis*), FM174388 for zebrafish (*Danio rerio*), AB465601 for Schlegel's black rockfish (*Sebastes schlegelii*), XM_007559717 for Amazon molly (*Poecilia formosa*), AB200968 for Japanese flounder (*Paralichthys olivaceus*), XM_010747018 for large yellow croaker (*Larimichthys crocea*), XM_008437864 for guppy (*Poecilia reticulata*), AM982800 for rainbow trout (*Oncorhynchus mykiss*), XM_012852921 for mummichog (*Fundulus heteroclitus*), Location: *Tn. Chr. 2*, 4493790:4496325 for green spotted pufferfish (*Tetraodon nigroviridis*), Location: *Fr. Scaffold_571*, 106000: 110000 for Fugu ([*Takifugu rubripes*](http://www.sciencedirect.com/science/article/pii/S0196978104005704)), M17706 for human (*Homo sapiens*), and M13926 for mouse (*Mus musculus*).


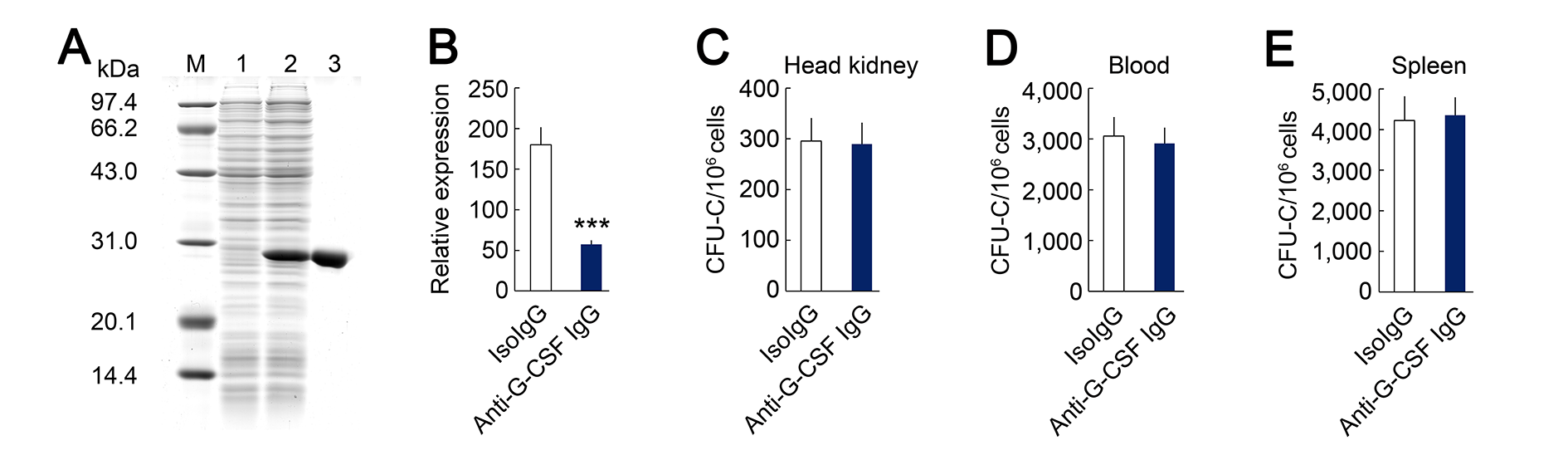


**Supplemental Figure 4. Effect of anti-G-CSF IgG on the CFU-Cs.** (A) Prokaryotic expression and purification of recombinant G-CSF. The proteins were kept tract using 15% SDS-PAGE. Lane M: protein marker; 1: before IPTG induction; 2: after IPTG induction; 3: purified recombinant protein. (B) The relative expression of G-CSF protein measured in plasma of ayu infected with *L. anguillarum* infection for 24 h after isoIgG or anti-G-CSF IgG treatment. (C-E) The number of CFU-Cs in cells from the head kidney, blood, and spleen of ayu treated with anti-G-CSF IgG after *L. anguillarum* infection for 24 h. Data are expressed as mean ± SEM. n = 5. **p*<0.05, ***p*<0.01, ****p*<0.001.


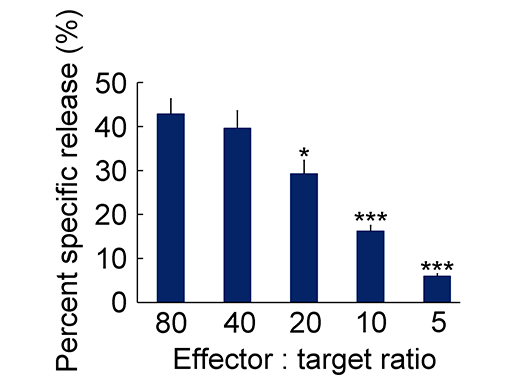


**Supplemental Figure 5.** *In vitro* cell-mediated cytotoxicity of PBL against allogeneic cells. It shows a hemoglobin release assay with allogeneic erythrocytes of wild environmental ayu. PBL was isolated from Zhemin No.1 ayu. Data are expressed as mean ± SEM. n = 5. **p*<0.05, ****p*<0.001.


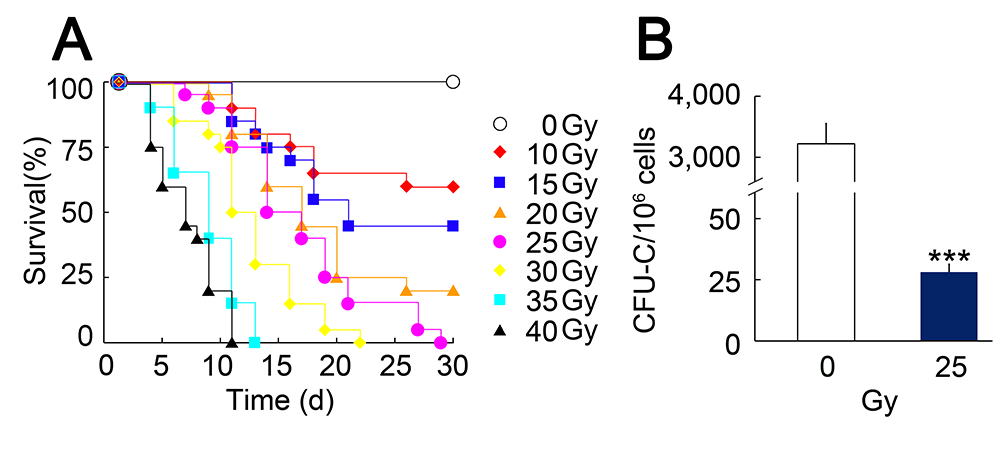


**Supplemental Figure 6. Determination of the minimum lethal dose of irradiation in ayu.** (A) Kaplan-Meier survival curves following graded doses of total body irradiation. n = 20. (B) The number of CFU-C in ayu head kidney cells 8 days after irradiation with 0 or 25 Gy. n = 5. ****p*<0.001.


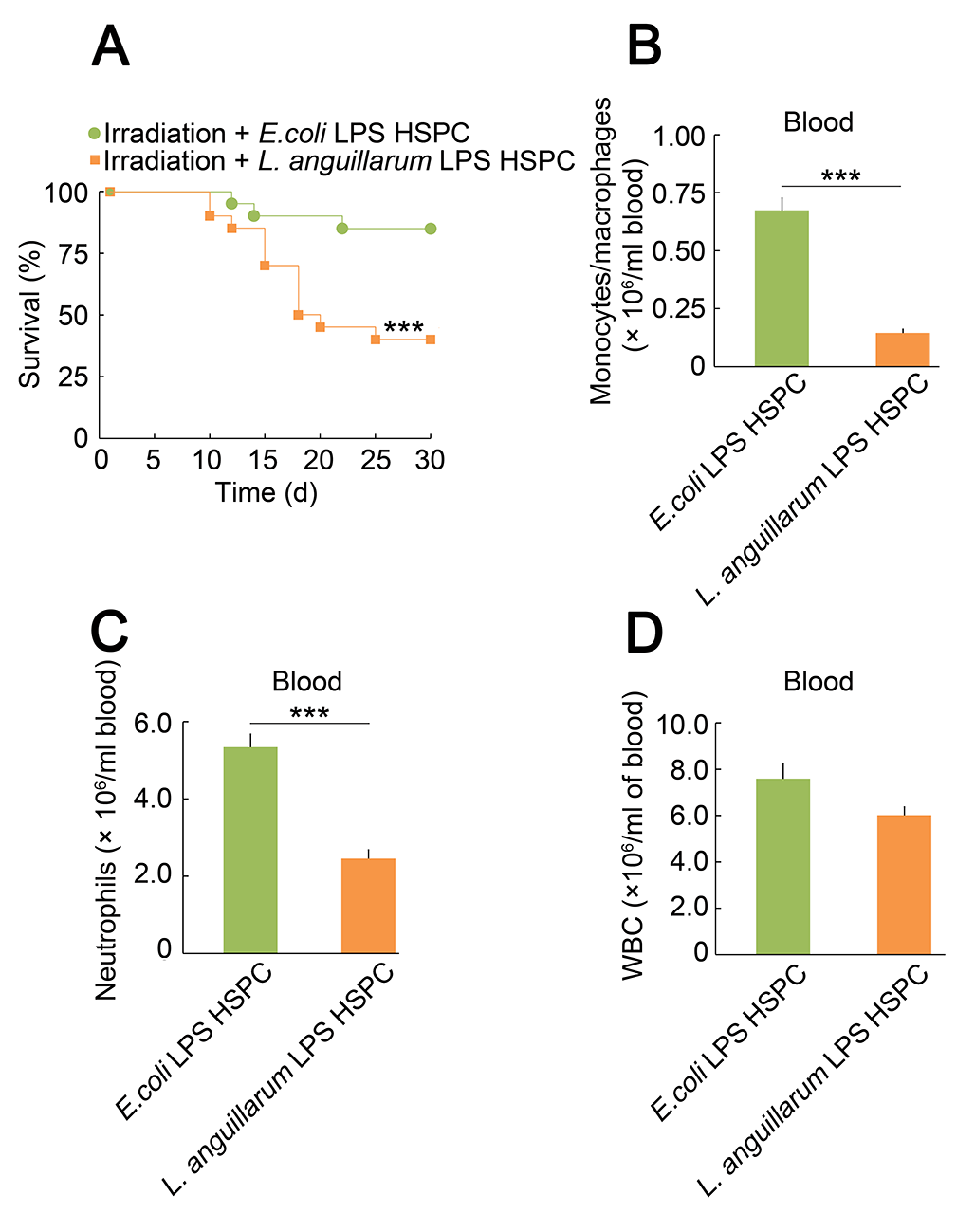


**Supplemental Figure 7. Survival rate and myeloid cell numbers of irradiated ayu treated with *E. coli* or *L. anguillarum* LPS HSPCs.** (A) Survival rate after transplantation of *E. coli* or *L. anguillarum* LPS HSPCs (1 × 10^5^ R3 cells). n = 20. HSPC transplantation affects the numbers of monocytes/macrophages (B), neutrophils (C), and total WBCs (D) in irradiated ayu. n = 5. **p*<0.05, ***p*<0.01, ****p*<0.001.
